# IoT-cloud based healthcare model for COVID-19 detection: an enhanced k-Nearest Neighbour classifier based approach

**DOI:** 10.1007/s00607-021-00951-9

**Published:** 2021-04-30

**Authors:** Rajendrani Mukherjee, Aurghyadip Kundu, Indrajit Mukherjee, Deepak Gupta, Prayag Tiwari, Ashish Khanna, Mohammad Shorfuzzaman

**Affiliations:** 1 Department of Computer Science and Engineering, University of Engineering and Management, Kolkata, India; 2 Department of Computer Science and Engineering, Brainware University, Kolkata, India; 3 Department of Computer Science and Engineering, Birla Institute of Technology, Mesra, India; 4 Maharaja Agrasen Institute of Technology, Delhi, India; 5 Department of Computer Science, Aalto University, Espoo, Finland; 6 Department of Computer Science, College of Computers and Information Technology, Taif University, Taif, 21944 Saudi Arabia

**Keywords:** Classifier, Cloud, COVID-19, Feature, Healthcare, IoT, 68

## Abstract

COVID - 19 affected severely worldwide. The pandemic has caused many causalities in a very short span. The IoT-cloud-based healthcare model requirement is utmost in this situation to provide a better decision in the covid-19 pandemic. In this paper, an attempt has been made to perform predictive analytics regarding the disease using a machine learning classifier. This research proposed an enhanced KNN (k NearestNeighbor) algorithm eKNN, which did not randomly choose the value of k. However, it used a mathematical function of the dataset’s sample size while determining the k value. The enhanced KNN algorithm eKNN has experimented on 7 benchmark COVID-19 datasets of different size, which has been gathered from standard data cloud of different countries (Brazil, Mexico, etc.). It appeared that the enhanced KNN classifier performs significantly better than ordinary KNN. The second research question augmented the enhanced KNN algorithm with feature selection using ACO (Ant Colony Optimization). Results indicated that the enhanced KNN classifier along with the feature selection mechanism performed way better than enhanced KNN without feature selection. This paper involves proposing an improved KNN attempting to find an optimal value of k and studying IoT-cloud-based COVID - 19 detection.

## Introduction

IoT-Cloud based healthcare predictive models play an important role in the detection of several diseases and provide better decisions to the users. It is necessary to propose such an IoT-cloud-based healthcare model for the detection of COVID-19. COVID-19 is mainly caused by Severe Acute Respiratory Syndrome Coronavirus 2 (SARS-CoV-2). It has been declared as a pandemic since March 2020, and according to WHO (World Health Organization), there are 21,409,133 active cases worldwide (December 2020). The virus mainly spreads by respiratory precipitations while coughing, sneezing, etc., and gets transmitted from person to person. While fever, cough is the primary symptoms of the disease, certain pre-existing medical conditions

(heart disease/diabetes/COPD/cancer) actually aggravate the disease’s outcome. Towards an effort to curb this disease and build a pandemic prepared healthcare system, this paper applies predictive data mining techniques on COVID - 19 data to understand the disease better.

Several researchers [5, 6, 8, 33, 35, 42, 43] have applied machine learning as well as deep learning algorithms on COVID - 19 data and derived fruitful insights. While some researchers used neural networks [5–7] or deep learning methods, others used regression [12], or classification algorithms [21, 24] to predict the prognosis of the disease. In this paper, we have explored the supervised learning algorithm KNN (*k* Nearest Neighbor) [14, 16–18] on seven COVID - 19 cloud datasets gathered across the world (Asia, Brazil, Mexico, etc.). The dataset sample size ranged from 5000 to 1.5 million. KNN algorithm was opted for because of its simplicity and ease of use. The experimentation demonstrated an enhanced KNN (*k* Nearest Neighbor) algorithm, which uses a *radical* mathematical function (square root / cube root / fourth root etc.) of the sample size of the dataset as the *k* value. The *k* is represented as ($\root n \of {N}$) where the radicand *N* is actually the sample size of the dataset (number of records present in the dataset) and $n~=~2,~3,~4$, etc. Thus, the *k* value is chosen dynamically during the runtime depending on the dataset size. In traditional KNN, the value of *k* is chosen arbitrarily or randomly [20, 25]. The enhanced KNN (eKNN) overcomes this shortcoming. Several performances indicating parameters (accuracy, precision, F1- score, error rate, etc.) [26, 27, 31, 32] were calculated to show the effectiveness of the proposed *eKNN*. The performance of *eKNN* algorithm was improved when used with ACO (Ant Colony Optimization) based feature selection mechanism [30]. For a fair comparison, C4.5 based FS mechanism was also investigated. The parameters were recalculated and compared with enhanced KNN without feature selection. Several graphical representations were shown for better performance visualization.

### Contribution

The paper contributed two noteworthy research questions -

***RQ1 - How can the KNN classifier choose the optimal value of k?***

The conducted literature survey did not show any experimentation regarding this shortcoming. To bridge this gap, this research proposed *eKNN* (Enhanced KNN) and overcame the limitations of traditional KNN. The newly proposed eKNN was applied on seven benchmark datasets gathered from the COVID cloud repository of different countries (Brazil, Mexico, etc.). This data analysis can act as a back-end of a healthcare model where IoT is used to develop the front-end interface.

***RQ2 - What is the effect of using the KNN classifier with feature selection mechanism?***

The proposed *eKNN* algorithm is used with a feature selection mechanism and made robust. Apart from leveraging ACO based feature selection mechanism, C4.5 based FS mechanism is also explored for a fair comparison. All the datasets were retested. Graphical illustrations are shown to visualize the effect.

### Organization

The paper is organized as follows: Section 2 underlines the related work, while Sect. 3 discusses the proposed methodology and working principle of the proposed *eKNN* algorithm. Section 4 describes the used dataset. Section 5 demonstrates the experimental setup and proves the fair comparison of the traditional KNN, *eKNN* with feature selection, and *eKNN* without feature selection. Finally, a conclusion is provided in Sect. 6 with future directions.

## Related work

Many researchers [22, 23, 36, 37, 39, 47–49] have been exploring machine learning techniques as well as deep learning techniques for health monitoring and tracking COVID - 19 over the cloud and IoT platforms. These studies can help in effective prediction and decision-making regarding the deadly disease. These experiments can help in early intervention, and thus it can eventually reduce the mortality rate.

Khanday et al. used traditional and ensemble machine learning classifiers for classifying textual clinical reports [14]. The authors found out that logistic regression and Naïve Bayes performed better than other ML algorithms by reaching 96.2% testing accuracy. Tiwari et al. [28] also proposed an unsupervised terminformer model to mine terms from the biomedical literature for COVID-19. Flesia et al. studied 2053 individuals with 18 socio-psychological variables and identified participants with elevated stress levels using a predictive machine learning approach [13]. Randhawa et al. focused on the COVID - 19 virus genome signature [15]. The authors combined machine learning and digital signal processing (MLDSP) for classifying COVID - 19 virus genomic sequence. Souza et al. applied several supervised ML algorithms (linear regression, decision tree, SVM, Gradient Boosting, etc.) on COVID positive patients and compared the outcome of each method [12]. Yan et al. studied 2799 patients of Wuhan and designed a prediction model using XGBoost to predict mortality [11]. SIR (Susceptible, Infected, and Recovered) model and machine learning were used for COVID - 19 pandemic forecasting by Ndiaye et al. [10]. Castelnuovo et al. studied Random Forest and indicated that decreased renal function is a potential cause of death in COVID patients [5]. Lalmuanawmaa et al. showed how AI could create less human interference in medical diagnosis [34]. Somasekar et al. applied neural networks for image classification of Chest X-ray [7, 45, 46]. Amar et al. applied regression on COVID data and predicted a number of infected people [8]. The authors collected data from Egypt. The average discharge time of COVID patients from the hospital was analyzed by Nemati et al., and this study [9] helped the hospital professionals to stay better prepared for the disease.

The application of KNN [19, 38, 41] and feature selection mechanism [1, 2] for medical diagnosis is also not new. In 2016, Li et al. conducted a study with EEG graphs regarding depression and found out that optimal performance is achieved using a combination of CFS (Correlation Features Selection) and KNN [3]. Remeseiro et al. applied the feature selection technique for medical applications. A case study was conducted on two medical applications with real-life patient data [4].

Inspired by this background work, this research gained motivation to pursue the implementation of machine learning approaches on COVID - 19 detection.

## Proposed methodology

In the first setup, the eKNN algorithm was applied without a feature selection mechanism on several datasets obtained from the COVID cloud. This back-end data analysis can be utilized to build an IoT-based front-end Covid screening system.

### Enhanced KNN (eKNN)

In the next step, the *eKNN* classifier was also applied on all seven datasets. Classification is a supervised ML method, and the role of a classifier is to map the input data into classes. Each object is classified into one group only. The KNN classifier was developed by Fix and Hodges in 1951 [19] and is an example of the simple classification algorithm. It can be used for both classification and regression. In KNN, distances (Manhattan, Euclidean, Minkowsky, Chebyshev, etc.) are calculated between the test sample and training data samples, and thus nearest neighbors are obtained. The neighbors are chosen from a set of training objects whose classes are already available. The test sample is assigned to the class of its nearest neighbor only.

### KNN representation

The KNN join of two sets *P* and *Q* is represented by $\{(p, \text {kNN}(p, Q)), \forall p \in P\}$ where p and q are two elements as $p \in P$ and $q \in Q$. k points (which are closest to p) are found from set Q in a dimensional space d.

The KNN algorithm suffers from a limitation and that is its dependency in choosing a proper value of parameter *k* (number of nearest neighbors). The performance of KNN algorithm hugely depends on this factor as the *k* value elects the number of neighbours which determines the class. In most of the cases, the *k* value is chosen randomly and this paper attempts to overcome this drawback.

In this research, the radical mathematical function (square root, cube root, fourth root etc.) is used to determine the *k* value dynamically during the computation time depending on the size of the dataset. The *k* is represented as ($\root n \of {N}$ ) where the radicand *N* is actually sample size of the dataset (number of records present in the dataset) and $n = 2, 3, 4$ etc. For example

- When the dataset contains around 5000 records (Dataset 1 in Table 1), the *k* value is set to $\root 2 \of {5000} \approx 71$. A small *k* value does not produce expected output because of noise/outlier (presented in Table 3) and because of that reason *k* value was not set to $\root 3 \of {5000} \approx 17$.
- But, when the dataset increases in size and contains 0.1 million (Dataset 3) / 0.25 million (Dataset 4) /0.5 million (Dataset 5) records, the *k* value is set to $\root 3 \of {N}$ . The value of *n* is increased from 2 to 3 because too high k value increased computation time heavily. For example, for Dataset 3, if *k* value is chosen as $\root 2 \of {100000} \approx 317$, then it costs huge increase in KNN algorithm running time (almost 2 times as presented in Table 3) and thus it creates poor performance. So, to avoid this performance degradation, the *k* value is set to $\root 3 \of {100000} \approx 47$ and the estimated computation time of KNN becomes manageable.
- Again, when the dataset becomes bigger with around 1 million (Dataset 6) or 1.5 million (Dataset 7) records, the *k* value is set to $\root 4 \of {N}$ . For example, for Dataset 6 the *k* value is set to $\root 4 \of {1000000} \approx 31$.
- In this *eKNN*, the value of *k* is kept odd, to avoid equal voting.

This choice of *k* is analytical and forms a basis of fair judgement, rather than choosing the *k* value arbitrarily. The data volume plays an important role is setting the *k* value and deciding the value of *k* in this manner will support application of KNN for large datasets. The previous steps are summarized below - 
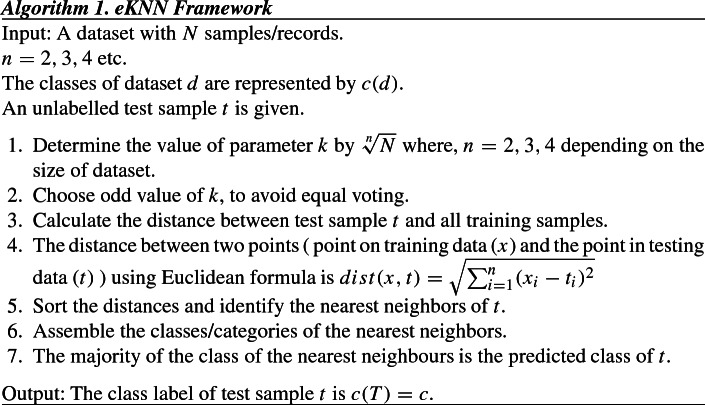


As the proposed *eKNN* classifier was applied on all the seven datasets under experimentation and obtained a confusion matrix. The matrix contains info about the actual and predicted value on classification. For each *eKNN* run, the accuracy, precision, recall, specificity, error rate, F1-score parameters were calculated from the confusion matrix. The resultant values are tabulated in Sect. 5.

### eKNN with ACO-based feature selection

In this second phase of implementation, the KNN algorithm was used with ACO based feature selection mechanism. Table 1 Dataset 1 has 8 features; Dataset 2, Dataset 6, and Dataset 7 each have 15 features; Dataset 3 and Dataset 4 each have 18 features while Dataset 5 has 19 features. It is explored whether ACO-based FS (Feature Selection) will bias the implementation potential of the proposed eKNN. If the original feature space is represented by size *S*, then the feature selection process’s goal is to select the optimal subset of features of size *s* ($s \le S$).

Ant colony optimization replicates ant’s food searching behavior pattern. As the ants move from one node to another, a chemical substance (pheromone) is deposited along the path. The pheromone trail helps other ants to find the food source following the shortest path. The pheromone evaporates at a certain rate resulting in the decay of less traversed paths. ACO is a probabilistic technique that ensures convergence and promotes rapid solution-finding. Because of these traits, ACO was given preference over others.

- The edges between the nodes guide the choice of next feature
- Amount of pheromone level is indicated by $\tau$
- The features which belong to the route with high level of pheromone are treated as the selected features.
- Selected feature subset is governed by -
  1 $$\begin{aligned} P_{ij}^n (t) = \frac{[\tau _{ij}(t)]^{\alpha } * [\eta _{ij}(t)]^{\beta }}{\sum _{u \in j}n[\tau _u(t)]^{\alpha } * [\eta _u(t)]^{\beta }} ~~~ if~i \in j^n \end{aligned}$$
  - Where $P_{ij}^n(t)$ is the probability of an ant at feature i moving to feature *j* at time instant *t*
  - *n* is number of ants / number of features
  - $j^n$ is set of potential features that can be present in temporal solution
  - $\tau _{ij}$ indicates amount of pheromone in edge (*i*, *j*)
  - $\eta _{ij}$ indicates heuristic value associated with edge (*i*, *j*)
  - All features have same value of $\tau ,~\eta$ initially
  - $\alpha> 0,~\beta > 0$ ($\alpha ,~\beta$ is determined experimentally and taken as $\alpha = 2,~\beta = 0.5$ in this study)
  - Pheromone evaporation rate is 5% in this study.
  - Stopping criteria is maximum number of iterations.

The basic operation of this ACO-based feature selection mechanism is depicted in Fig. 4. After applying the ACO-based FS technique, among 8 features of Dataset 1, 5 features were selected (detailed in Table 2). Dataset 2, Dataset 6, and Dataset 7 have the same features, and 10 features were selected out of 15. Dataset 3 and Dataset 4 have the same features, and 11 out of those 18 features got selected. Dataset 5 initially had 19 features, and after the FS mechanism, 11 got selected. The selected features are tabulated in Table 2. The eKNN algorithm was applied on the reduced datasets, and accuracy, precision, recall, specificity, error rate, F1-score parameters were recalculated for each dataset. The calculated values are summarized in Sect. 5. After applying the eKNN classifier with ACO-based FS mechanism, C4.5 based FS mechanism was also explored to evaluate which FS mechanism is a better performer. The result comparison is tabulated in the next section.

## Dataset description

The experimentation involved seven standard COVID datasets of different sizes and origins. Table 1 represents the datasets in ascending order of their size. For convenience, we named the datasets with Dataset 1, Dataset 2, and Dataset 3, etc.

**Table 1:** Subject COVID- 19 Datasets for experimentation

Dataset name	Sample size (No. of Records)	Number of features	Origin
Dataset 1	5000	8	Kaggle
Dataset 2	50000	15	https://ourworldindata.org/ [Jan 22,2020 to March 30,2020] [Cross Country dataset]
Dataset 3	100000	18	Released by Brazilian government https://coronavirus.es.gov.br/painel-covid-19-es [6/1/2020 to 10/8/2020 from Brazil]
Dataset 4	250000	18	Released by Brazilian government https://coronavirus.es.gov.br/painel-covid-19-es [6/1/2020 to 21/12/2020 from Brazil]
Dataset 5	500000	19	Released by Mexican government https://www.gob.mx/salud/documentos/datos-biertos-152127
Dataset 6	1000000	15	https://ourworldindata.org/ [April 1,2020 to April 7,2020] [ Cross Country dataset]
Dataset 7	1500000	15	https://ourworldindata.org/ [August 1,2020 to August 7,2020] [ Cross Country dataset]

**Table 2:** Selected subset of features after ACO based FS mechanism

Dataset name	Original no. of features	Selected no. of features	Selected features after ACO based FS
Dataset 1	8	5	Age, gender, country, date, diabetic
Dataset 2, Dataset 6 and Dataset 7	15	10	Age, country, date, heart comorbidity, pregnancy, COPD, diabetic, cough, diarrhea, obesity comorbidity
Dataset 3 and Dataset 4	18	11	Gender, cough, sore throat, breathing difficulty, renal comorbidity, headache, fever, serology result, smoking, ICU, asthma
Dataset 5	19	11	Gender, tobacco, pneumonia, hypertension, COPD, diabetic, cough, diarrhea, pregnancy, sore throat, renal comorbidity

The first dataset is collected from Kaggle, and it has 5000 records (both COVID positive and COVID negative cases). The second dataset is a cross-country dataset (Our World in Data COVID-19 cloud dataset [29]) focusing on COVID - 19 testing data obtained from https://ourworldindata.org/ till March 2020 starting from Jan 22, 2020. Dataset 6 and Dataset 7 are also gathered from ’Our World in Data COVID-19 dataset’ [29], but they represent different time frames. Dataset 6 is from April 1, 2020, to April 7, 2020, and it contains 1 million records indicating massive outbreaks across the world rapidly. Dataset 7 is collected based on August 1, 2020, to August 7, 2020, and it contains 1.5 million records. Dataset 3 is obtained from the Brazilian government’s cloud (https://coronavirus.es.gov.br/painel-covid-19-es), and it contains 0.1 million records from 6/1/2020 to 10/8/2020. Dataset 4 is also from Brazil, and it contains a total of 0.25 million cases reported from 6/1/2020 to 24/12/2020. Dataset 5 contains 0.5 million records and is released by Mexican government (https://www.gob.mx/salud/documentos/datos-abiertos-152127).

**Fig. 1:**
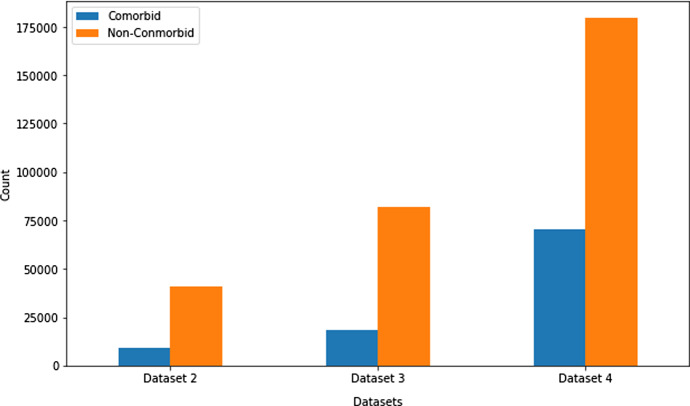
Patients with comorbidity and non-comorbidity

**Fig. 2:**
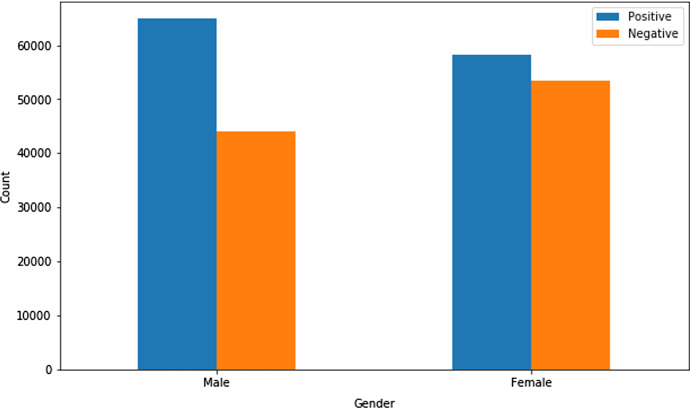
Gender distribution of COVID positive and COVID negative patients from dataset 4

**Fig. 3:**
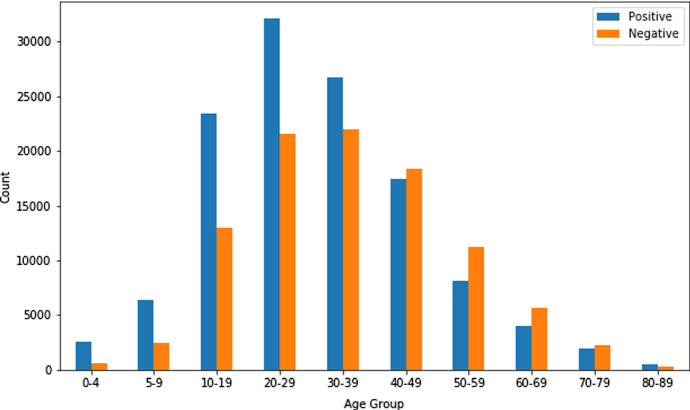
Age group distribution of COVID positive and COVID negative patients from dataset 4

All the datasets contain several features like age, gender, country, heart disease, COPD, diabetes, pregnancy, smoking habits, etc. Three random datasets (Dataset 2, Dataset 3, and Dataset 4) were chosen to measure the presence of comorbidity, and Fig. 1 represents the count of patients with Comorbidity and Non-Comorbidity. One random dataset (Dataset 4) was chosen to judge the age and gender distribution. Figure 2 depicts the gender distribution and Fig. 3 portrays the age group distribution from Dataset 4 for COVID positive and negative patients. The count of COVID positive male patients was slightly higher than COVID positive female patients, according to Fig.2 as obtained from Dataset 4.

**Fig. 4:**
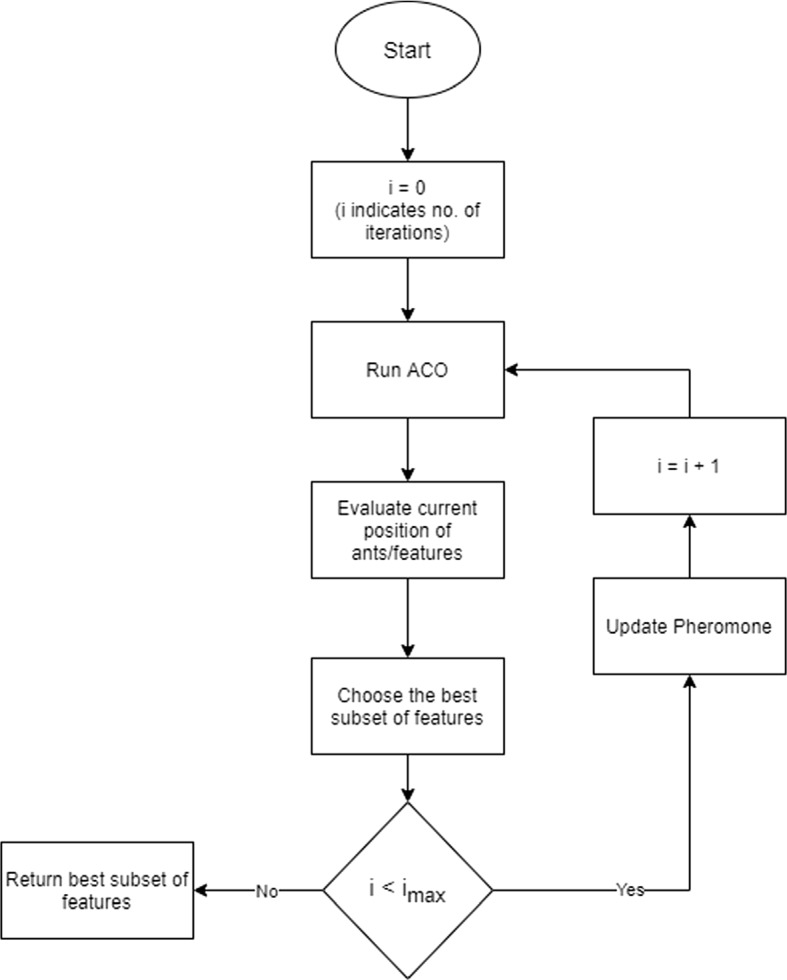
Framework of ACO based feature selection mechanism

## Experimental results and discussion

### Data preprocessing

The transformation of raw data into a meaningful format is known as data preprocessing. Data Quality plays a vital role in determining the experimental results. All the seven subject datasets were pre-processed and cleaned. As part of the data cleaning activity, duplicate records were deleted. Several records had certain fields as NULL (age in Dataset 2, Serology Result in Dataset 4, Blood Pressure in Dataset 5, etc.). The NULL values were replaced with an average value of that particular field. Only very few records had inconsistent/junk characters in many fields, so such records are discarded.

For all the 7 datasets, the KNN algorithm was initially implemented using random values of *k*. The choice of the *k* parameter is made arbitrarily, and the confusion matrix is obtained for each case.

### Evaluation measures

**Fig. 5:**
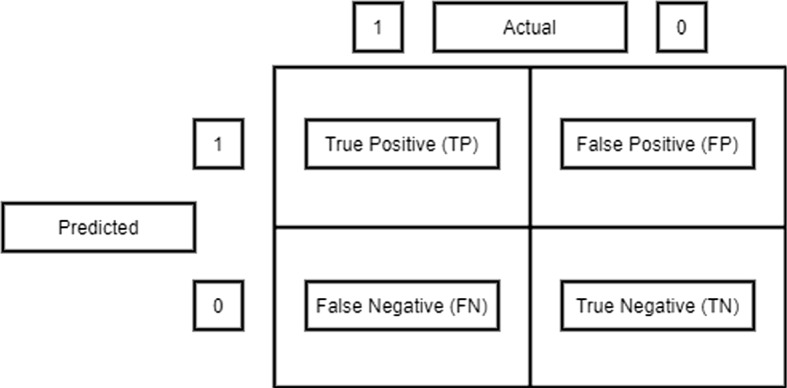
Illustration of confusion matrix

The confusion matrix helps to measure the performance of a classifier. It is a matrix of two dimensions (Actual and Predicted), and the dimensions have TP(True Positive), TN(True Negative), FP(False Positive), and False Negative(FN) as presented in Fig. 5. True positive are the cases where the predicted value is yes (having the disease), and the patients really have the disease. True negative is when the predicted value is no (not having the disease), and the patients really do not have the disease. False-positive are the cases where the predicted value is yes (having the disease), but the patients actually do not have the disease. False-negative are the cases where the predicted value is no (not having the disease), but the patients have the disease. Several performance indicators of a classifier are derived from this matrix. For example -

2 $$\begin{aligned} \mathrm Accuracy&= \frac{TP+TN}{TP+TN+FP+FN} \end{aligned}$$

3 $$\begin{aligned} \mathrm Precision&= \frac{TP}{TP+FP} \end{aligned}$$

4 $$\begin{aligned} \mathrm Error~Rate&= \frac{FP+FN}{TP+TN+FP+FN} \end{aligned}$$

**Table 3:** Performance parameters with different neighbors (k value) for KNN and eKNN

Dataset name	Size (N)	Basis of k value choice	k value	Accuracy (in%)	Precision (in %)	Specificity (in %)	Recall (in %)	F1 score (in %)	Error rate (in %)	Time taken (in %)
Dataset 1	5000	Randomly	37	75.6	88.5	85.4	65.2	75.4	9.5	6.45
		$\root 2 \of {N}$	71	87.9	85.4	84.3	73.7	76.9	5.6	8.20
		$\root 3 \of {N}$	17	68.1	83.6	80.5	63.2	72.1	13.2	4.26
Dataset 2	50000	Randomly	25	80.2	82.1	80	76.5	76.3	7.5	9.57
		$\root 3 \of {N}$	37	89.9	85.4	84.3	77.8	76.4	7.8	10.03
		$\root 2 \of {N}$	223	91.2	87.6	85.3	80.4	78.6	6.5	21.5
Dataset 3	0.1 million	Randomly	35	86.5	84.3	83	77.6	75.4	7.4	10.2
		$\root 3 \of {N}$	47	91.5	84.1	83.5	77.8	75.3	7.5	11.5
		$\root 2 \of {N}$	317	92.5	85.8	84.6	79.8	78.2	6.2	25.4
Dataset 4	0.25 million	Randomly	35	72.24	81.11	86.1	66.7	70.2	15.6	12.5
		$\root 3 \of {N}$	63	86.97	80.32	84.3	75.9	78.3	7.2	16.5
Dataset 5	0.5 million	Randomly	27	69.4	85.1	84.5	57.6	65.3	18.7	15.7
		$\root 3 \of {N}$	79	87.8	82.3	81.1	76.8	75.8	7.54	20.5
Dataset 6	1 million	Randomly	63	85.4	80.7	80.5	74.2	73.2	8.9	24.6
		$\root 3 \of {N}$	101	89.78	81.2	79.5	74.5	70.5	10.2	30.6
		$\root 4 \of {N}$	31	89.5	83.2	80.5	75.8	72.1	6.5	20.45
Dataset 7	1.5 million	Randomly	71	85.5	82.1	81.2	75.3	73.2	10.6	25.6
		$\root 3 \of {N}$	113	91.5	79.7	75.4	73.5	72.6	15.5	40.3
		$\root 4 \of {N}$	35	90.6	85.4	83.2	77.6	75.3	7.8	30.5

The existing KNN algorithm was experimented (with random *k* value) on all the datasets, and the resultant confusion matrix yielded several performance parameters. The calculated performance metrics are - Accuracy, Precision, Specificity, Recall, F1 score, Error Rate. In each case, the computational time to validate the algorithm is also noted. The results are listed in Table 3 for respective *k* values for corresponding datasets. After this initial experiment, the proposed eKNN algorithm is validated, and the k value is chosen based on a radical mathematical function. Table 3 contains all the recalculated performance parameters after the *eKNN* implementation of each dataset.

**Fig. 6:**
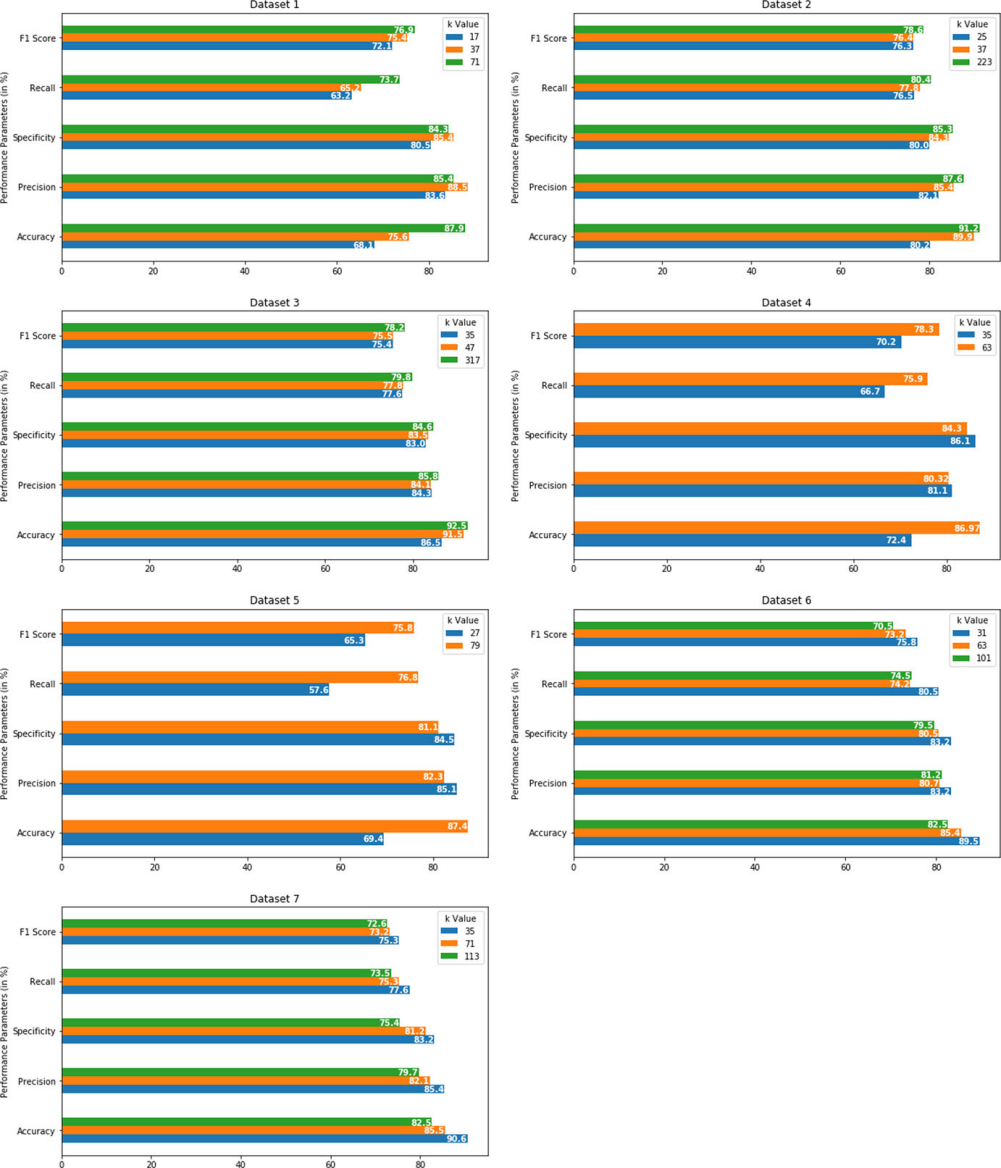
Performance parameters of KNN and eKNN classifier with different k values

Figure 6 contains a graphical depiction of these parameters for different *k* values.

### Experimental analysis and findings

From the obtained experimental results (Table 3), it can be summarized -

- In terms of Accuracy, F1 score, Error Rate, Computation time, etc., performance indicator, the eKNN algorithm performed significantly better than the ordinary KNN algorithm.
- Results indicated that for Dataset 1, when k was chosen randomly (k = 37), the obtained accuracy was only 75.6%. But by the use of eKNN, when k was chosen as $\root 2 \of {N}$ (in this case N = 5000, so k = 71), the obtained accuracy increased to 87.9%. It was also observed that the computation time of eKNN (8.2 seconds) was negligibly higher than ordinary KNN (computation time of 6.45 seconds). Also, the error rate became lower (5.6%) in comparison to normal KNN (an error rate of 9.5%). So, eKNN showed manifold improvement.
- For Dataset 1, k value was chosen as $\root 2 \of {N}$ , instead of $\root 3 \of {N}$ because too low k value cannot handle outliers/noise. Results indicated severe damage in accuracy (only 68.1%), when k value became too small ($k = \root 3 \of {N} = 17$).
- The essence of this eKNN algorithm is that it dynamically chooses the k value depending on the dataset’s size. While k is presented as $\root n \of {N}$, the radical mathematical function (square root / cube root / fourth root etc.) varies according to sample size (N) of the dataset and thus n = 2, 3, 4 etc. This became very useful when the dataset size increased compared to Dataset 1 (only 5000 records). Dataset 2 and Dataset 3 have 50,000 and 0.1 million records, respectively. So for both of these datasets, k was chosen as $\root 3 \of {N}$ resulting in high accuracy of 89.9% and 91.5%, respectively (Table 3).
- For both Dataset 2 and Dataset 3, if k value was taken as $\root 2 \of {N}$, instead of $\root 3 \of {N}$  then the k value became very high, resulting in approximately double computation time while showing a very slight increase in accuracy and a minor decrease in error rate. For example, for Dataset 3, when $k = \root 2 \of {N} = 317$ was chosen, accuracy turned out as 92.5%, error rate appeared as 6.2% and the computation time became 25.4 seconds. But for the same dataset, when $k = \root 3 \of {N} = 47$ was chosen, accuracy turned out as 91.5% (very negligible decrease), error rate appeared as 7.5% (very minor increase) and the computation time became 11.5 seconds (less than half). So, high accuracy is preserved with very reasonable computation time, and thus these results supported the avoidance of too high a k value.
- For Dataset 4 (0.25 million) and Dataset 5 (0.5 million), the k value of $\root 3 \of {N}$ gave very high accuracy of 86.9% and 87.8% respectively. Even though the Precision parameter varied very slightly, the Recall and F1 score parameters also improved both datasets (Table 3).
- Results also indicated that if k value is taken too small, then accuracy decreases drastically. For example, for Dataset 5 (sample size 0.5 million), if the k value is set to 27, ($k = \root 4 \of {N} = 27$), then the obtained accuracy falls down to 69.4%. It was 87.8% when k was set to 79 ($k = \root 3 \of {N} = 79$).
- However, as the datasets became larger in size, the fourth root of radicand usage became necessary. Because too high a k-value will increase the computation time of the algorithm while showing minor accuracy improvement. Results of Dataset 6 (1 million) and Dataset 7 (1.5 million) indicated this. In both cases, high accuracy was obtained at $k =\root 4 \of {N}$. While Dataset 4 yielded an accuracy of 89.5%, Dataset 7 generated an accuracy of 90.6%. The algorithm’s computation time was also reasonable for both datasets (20.45 seconds and 30.5 seconds, respectively). But slight increment in accuracy (Dataset 6: 89.78%, Dataset 7: 91.2%) and a huge increment in computation time (Dataset 6: 30.6 seconds, Dataset 7: 40.3 seconds) were observed if the cube root of radicand was used to determine k value. Thus, the necessity of using $k =\root 4 \of {N}$ instead of $k =\root 3 \of {N}$ is reinforced for larger datasets.

**Table 4:** Comparison of performance parameters for KNN, eKNN, eKNN with ACO based FS and eKNN with C4.5 based FS mechanism

Dataset name	Performance metrics	kNN	eKNN	eKNN with ACO	eKNN with C4.5
Dataset 1	Accuracy (in %)	75.6	87.9	95.4	92.1
	computation time (in Seconds)	6.45	8.2	8.5	8.5
Dataset 2	Accuracy (in %)	80.2	89.9	96.7	93.2
	computation time (in Seconds)	9.57	10.3	11.5	11.43
Dataset 3	Accuracy (in %)	86.5	91.5	96.4	95.3
	computation time (in Seconds)	10.2	11.5	12.1	12.5
Dataset 4	Accuracy (in %)	72.24	86.97	93.55	94.7
	computation time (in Seconds)	12.5	16.5	17.6	17.4
Dataset 5	Accuracy (in %)	69.4	87.8	95.4	96.3
	computation time (in Seconds)	15.7	20.5	24.3	27.5
Dataset 6	Accuracy (in %)	85.4	89.5	95.3	92.5
	computation time (in Seconds)	24.6	20.45	25.45	26.6
Dataset 7	Accuracy (in %)	85.5	90.6	97.5	93.2
	computation time (in Seconds)	25.6	30.5	35.42	32.11

**Fig. 7:**
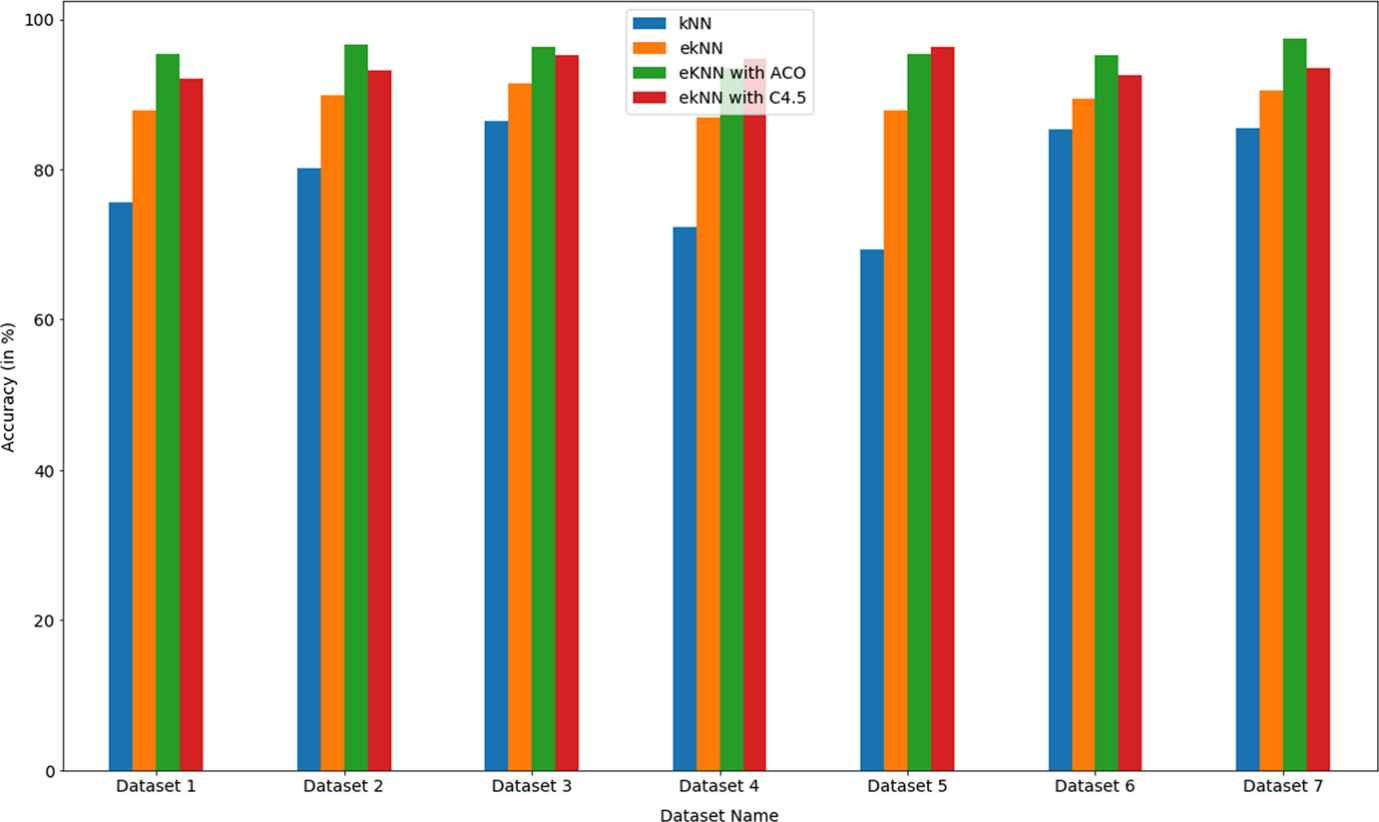
Comparison of accuracy values between KNN, eKNN and eKNN with FS mechanism

**Fig. 8:**
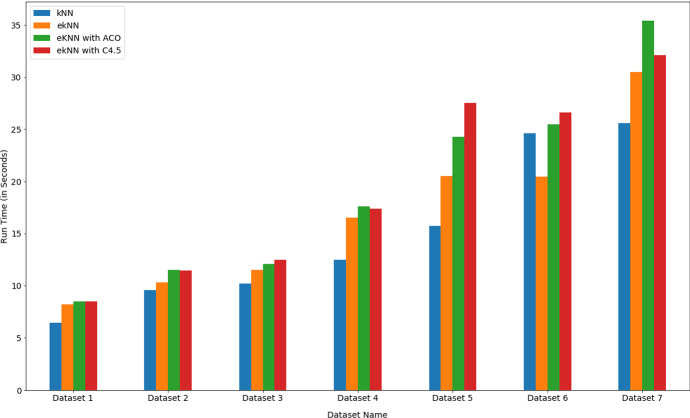
Comparison of computation time values between KNN, eKNN and eKNN with FS mechanism

Thus, the *eKNN* algorithm showed a judicial approach in choosing the *k* value instead of choosing it randomly. The *k* value was dynamically determined from the sample size of the dataset using the sample size as radicand, and this formed a logical-mathematical construct instead of arbitrarily chosen *k* value.

### Results on eKNN with feature selection

In the second phase of experimentation, as the *eKNN* algorithm was augmented with ACO-based feature selection mechanism, number of feature selected by each subject dataset got reduced. As described in Sect. 4, the features which belong to the route with a high level of pheromone were treated as selected features. According to Table 2, after applying ACO-based FS for maximum iterations, 5 features are selected of Dataset 1, while Dataset 2, Dataset 6, and Dataset 7 are reduced to 10 features each. On the other hand, Dataset 3, Dataset 4, and Dataset 5 selected 11 features from the initial 19 features. Table 2 enlists the names of all the selected features per dataset. For a fair comparison, the eKNN was also tested with C4.5 based FS. The performance parameters were computed.

The *eKNN* algorithm was applied on all the reduced datasets, and significant improvement in accuracy value was observed. There was a negligible increase in computation time because of feature selection implementation. However, the benefit in terms of accuracy was significant. Table 4 presents the findings. From the results as detailed in Table 4, it is evident that -

- In all the datasets, *eKNN* with FS showed improved accuracy over eKNN without FS. The improvement is in the range of 5% to 7% (see Table 4).
- Among 7 datasets, in 5 cases *eKNN* with ACO-based FS mechanism showed significant improvement in accuracy value over *eKNN* without FS mechanism.
- In the case of two datasets (Dataset 4 and Dataset 5), *eKNN* with C4.5 based FS mechanism showed the highest accuracy values.
- The computation time of eKNN with FS mechanism was negligibly higher than *eKNN* without FS. The increment time was remarkably minor (in the range of 0.3 seconds to 6 seconds only)(Table 4). Only in one exceptional case for Dataset 6, the computation time of KNN was higher than eKNN (maybe because of the high *k* value chosen randomly).
- In two datasets (Dataset 2 and Dataset 7), *eKNN* with ACO-based FS mechanism showed higher computation time than *eKNN* with C4.5 based FS. In the rest of the datasets, *eKNN* with C4.5 based FS took a higher time to compute.

Thus, from the results, it is very much clear that *eKNN* with ACO-based FS performed consistently better than all other experimented techniques. The performance parameter comparison is depicted in Figs. 7 and 8 for better visualization.

Comparison of results with previous studies also showed a very promising prospect. While the mean accuracy value for eKNN with ACO based FS mechanism came as 95.75%, studies conducted by De Souza et al. produced an accuracy of 85% [12] after the application of KNN classifier on the same COVID datasets. Also, it is noteworthy that while CPDS (COVID-19 Patients Detection Strategy) (developed in October 2020) based on a hybrid feature selection mechanism (HFSM) gave a promising result (93% accuracy), this eKNN based on ACO based feature selection mechanism produced higher accuracy of (95.75%). So, it is obvious that the proposed eKNN algorithm can be applied for COVID - 19 data analysis with very high performance.

## Conclusion

COVID - 19 is a highly contagious disease caused by the newly found coronavirus, and it has jolted our healthcare system. Even though most of the people infected by this disease recover without hospitalization or special medical attention, it has become fatal in many cases. While some people recovered from the disease soon, some people with an underlying health condition (diabetes, heart condition) had passed away. Towards an effort to navigate this enigma, this paper attempts to build an IoT-cloud-based healthcare model for COVID-19 detection using several datasets.

In this paper, we tried to propose an IoT-cloud-based healthcare predictive model to detect COVID-19 using eKNN. A novel enhanced KNN classifier (eKNN) is proposed, which chooses the k value using a radical mathematical function instead of choosing it randomly. The newly designed eKNN algorithm has been experimented on seven COVID - 19 benchmark cloud datasets from different countries (Brazil, Mexico, etc.). This classifier can act as a backend to an IoT-based frontend COVID screening system, and it can promote the processing of large datasets in reasonable time with high computational accuracy. The experiment showed that the eKNN algorithm with an ACO-based FS mechanism generated the best performance. The proposed eKNN can be beneficial to predict the outcome of the disease.

In the future, the work can be extended using weighted KNN or some other feature selection mechanism apart from ACO or C4.5 based mechanism. The work can also be extended for larger COVID datasets (gathered cumulatively over a wide time frame) in the big data domain using Hadoop / Map Reduce approach. This proposed classifier can perform better for disease detection to fight the disease and forecast possible outcomes.
